# Perceived ethnic discrimination and depressive symptoms: the buffering effects of ethnic identity, religion and ethnic social network

**DOI:** 10.1007/s00127-016-1186-7

**Published:** 2016-02-12

**Authors:** Umar Z. Ikram, Marieke B. Snijder, Matty A. S. de Wit, Aart H. Schene, Karien Stronks, Anton E. Kunst

**Affiliations:** Department of Public Health, Academic Medical Center, University of Amsterdam, P.O. Box 22660, 1100 DD Amsterdam, The Netherlands; Public Health Service Amsterdam, Epidemiology and Health Promotion, Nieuwe Achtergracht 100, 1018 WT Amsterdam, The Netherlands; Department of Psychiatry, Radboud University Medical Center, P.O. Box 9101, 6500 HB Nijmegen, The Netherlands; Donders Institute for Brain, Cognition and Behavior, Radboud University Nijmegen, P.O. Box 9109, 6500 HB Nijmegen, The Netherlands

**Keywords:** Perceived ethnic discrimination, Depressive symptoms, Ethnic identity, Ethnic social network, Religion

## Abstract

**Purpose:**

Perceived ethnic discrimination (PED) is positively associated with depressive symptoms in ethnic minority groups in Western countries. Psychosocial factors may buffer against the health impact of PED, but evidence is lacking from Europe. We assessed whether ethnic identity, religion, and ethnic social network act as buffers in different ethnic minority groups in Amsterdam, the Netherlands.

**Methods:**

Baseline data were used from the HEalthy Living In a Urban Setting study collected from January 2011 to June 2014. The random sample included 2501 South-Asian Surinamese, 2292 African Surinamese, 1877 Ghanaians, 2626 Turks, and 2484 Moroccans aged 18–70 years. Depressive symptoms were assessed using the Patient Health Questionnaire-9. PED was measured with the Everyday Discrimination Scale. Ethnic identity was assessed using the Psychological Acculturation Scale. Practicing religion was determined. Ethnic social network was assessed with the number of same-ethnic friends and amount of leisure time spent with same-ethnic people.

**Results:**

PED was positively associated with depressive symptoms in all groups. The association was weaker among (a) those with strong ethnic identity in African Surinamese and Ghanaians, (b) those practicing religion among African Surinamese and Moroccans, (c) those with many same-ethnic friends in South-Asian Surinamese, Ghanaians, and Turks, and (d) those who spend leisure time with same-ethnic people among African Surinamese and Turks.

**Conclusions:**

Ethnic identity, religion, and ethnic social network weakened the association between PED and depressive symptoms, but the effects differed by ethnic minority group. These findings suggest that ethnic minority groups employ different resources to cope with PED.

## Background

Discrimination is a social phenomenon that manifests itself in different forms in our contemporary society [9–11]. One form is perceived ethnic discrimination (PED), which represents the day-to-day experiences of overt and subtle acts of unfair treatment because of ethnic background [8, 11]. A survey indicated that ethnic discrimination tends to be widespread across Europe, with around 30 % of the ethnic minorities reported being discriminated against on grounds of ethnic background [12]. A Dutch report suggested a higher figure, around 40–50 %, and indicated that ethnic minorities mostly experience discrimination in the public space and in the labour market [14]. A 1991 qualitative study suggested that most African Surinamese women in Amsterdam experienced discrimination in the media, public space, and at work and school [16]. They were confronted with group-based stereotypes (e.g., lack of discipline, language deficit, low education, single mother). This was largely confirmed in a more recent qualitative study among 2nd-generation Surinamese and Moroccan adults (unpublished, conference presentation [17]).

Perceived ethnic discrimination is considered a chronic stressor, with growing evidence indicating that PED is positively associated with adverse physical and mental health outcomes among ethnic minority groups [8, 18–20]. Evidence seems to be most consistent with depressive symptoms, suggesting that higher PED is associated with more depressive symptoms across ethnic minority groups [19, 20]. Although it is pivotal to tackle discrimination itself, assessing which psychosocial factors weaken the association between discrimination and depressive symptoms enhances our understanding on how ethnic minority groups cope with PED [21, 22]. This might help understand why some people are more resilient to PED than others.

Previous research on psychosocial factors as potential buffers against PED has yielded mixed results. For example, a meta-analysis found that social support and group identification did not modify the association between discrimination and mental health outcomes [20]. A 2009 review reported mixed findings for racial identity as a buffer for the association between discrimination and health—in some studies racial identity actually tended to exacerbate the association [21]. This review also found that social support generally did not act as a buffer [21]. However, most studies had a relatively small sample consisting of young adults (mainly students), drawn with convenience sampling. Further, the majority of the studies were conducted in the United States (US)—mainly with African-Americans. European research on this topic is urgently needed. The relatively recent influx of migrants from across the world has dramatically changed the European demographics. Further, given the important socio-historical differences (e.g., migration history, countries of origin), findings from the US may not readily be applied to European-based ethnic minority group, who might experience and cope with discrimination differently than those living in the US [16].

In the present study, we focus on three potential psychosocial factors that are particularly relevant to the lives of these ethnic minority groups: ethnic identity, religion, and ethnic social network. Ethnic identity is defined as “the subjective sense of ethnic group membership that involves self-labelling, sense of belonging, preference for the group, positive evaluation of the ethnic group, ethnic knowledge, and involvement in ethnic group activities” [23, pp. 225, 24]. We hypothesise that strong ethnic identity weakens the association between PED and depressive symptoms. This might occur through taking pride in being a member of an ethnic group, which might buffer against the effects of PED [25, 26]. Strong ethnic identity may also create awareness of the socio-cultural history of the ethnic group, enabling individuals to adequately distinguish whether discrimination is directed at them personally, or at their ethnic group as whole [22]. Evidence suggests that attributing discrimination to the ethnic group instead of personal characteristics yields psychological benefits, as it prevents self-blame, personal devaluation and low self-esteem [10, 21].

Religion might weaken the association between PED and depressive symptoms. This may occur through its spiritual and social support component [27, 28]. Spirituality may enable an individual to acquire and employ different religious-specific coping styles (e.g., praying, seeking support from religious peers, accepting one’s fate), which may help to deal with stressors [29]. Research has shown that spirituality might have a beneficial impact on mental health, as it enables an individual to control feelings of anger and resentment [30]. In addition, religious institutions may provide professional social support and guidance in social and judicial affairs such as discrimination [28]. A study among African Americans showed that church-based social support buffered the association between racism and anxiety symptoms [31]. So far, very few studies have examined the buffering effects of religion on the association between PED and health. A 2008 Dutch report indicated ethnic minority groups were more religious and visited more often religious gatherings than ethnic Dutch; the highest rates were observed among Turks and Moroccans [32].

Ethnic social network reflects the presence of same-ethnic people within one’s social network. It is important to note that despite the close relationship between social support and social network, these constructs are different in that the former entails the quality of the social support one receives whereas the latter represent the extent and size of one’s network (i.e., quantity) [33]. We hypothesize that a large ethnic social network would weaken the association between PED and depressive symptoms. Ethnic social network promotes connectedness among same-ethnic people. This may not only help establish supportive and sustainable relationships, but also provides an opportunity to share personal experiences with those who might have experienced discrimination themselves [21]. A recent study found that family support moderated the association between discrimination and depression among Asian Americans [34]. An ethnic social network may also enable individuals to be involved in ethnic social activities, distracting from negative feelings, and providing positive interactions and experiences with same-ethnic people instead [35].

To test our hypotheses, we used a large population-based sample of adults from the five largest ethnic minority groups living in a medium-sized European city. For each ethnic minority group, we assessed whether ethnic identity, religion, and ethnic social network weakened the association between PED and depressive symptoms. The ethnic minority groups included in our study differ from each other in various ways (see Box 1), so we expect the buffering effects to differ by ethnic minority group. By investigating the potential buffering effects of these psychosocial factors in different ethnic minority groups in a European context, we could gain a broader understanding of the coping resources employed by ethnic minority groups against PED.

**Box 1:** Socio-historical information on the ethnic minority groups included in this study

*South Asian Surinamese* South-Asian Surinamese arrived in the Netherlands in the 1960–1970s, after the independence of Suriname from the Netherlands [4, 5]. The most important reason of migration was the unstable political and economic situation in Suriname. The ancestors of South-Asian Surinamese were originally from the Northern parts of India (e.g., Bihar), and worked as contract-workers in Suriname. Although Indian labourers were intended to stay temporarily, many decided to settle permanently in Suriname [4]. South-Asian ethnic populations are living across Europe, especially in the United Kingdom (UK) and the Scandinavian countries. *African Surinamese* African-Surinamese migrated from Suriname to the Netherlands, along with the South-Asian Surinamese [4, 5, 8]. They had similar reasons of migration as South-Asian Surinamese. They are descendants of West Africans who were brought to Suriname during the slave trade in the 18th and 19th century. African-Caribbean populations with similar socio-historical background can also be found in other European countries (e.g., UK and France) and the United States. In 2015, Surinamese people^a^ comprise 2 % of the general population and 17 % of total non-Western migrant population^b^ in the Netherlands [3]. In Amsterdam, these figures are 8 and 23 %, respectively [6]. Of the Surinamese living in the Netherlands, it is estimated that 45 % has South-Asian origin and 39 % African origin [15]. There is considerable religious diversity among Surinamese: 35 % Christian, 25 % Hindu, and 10 % Muslim (and 30 % non-religious) [15]. *Ghanaians* Ghanaians migrated to the Netherlands in two phases [1, 2]. In the first phase in the 1970s and 1980s many migrated because of socioeconomic reasons. During the second phase in the early 1990s was due to the unstable political situation in Ghana, drought, and the expulsion of many Ghanaians from Nigeria. There are also large Ghanaian communities in the UK and Germany. In the Netherlands, Ghanaian people comprise 0.1 % of the general population and 1 % of the total non-Western migrant population [3]. The figures were 1.5 and 4 %, respectively, in Amsterdam [6]. Most Ghanaians are Christian, but some are Muslim. *Turks and Moroccans* Migrants from Turkey and Morocco were recruited by the Dutch government in the 1960–1970s as temporary guest workers, to fill the labor shortages in unskilled occupations [2, 7]. However, the majority of the migrants decided to settle and brought their spouses and children to the Netherlands. Currently, Turks and Moroccans are the largest ethnic minority groups in many European countries (e.g., Spain, France, Germany). Turkish and Moroccan people separately account for around 2 % of the general population and 19 % of the total non-Western migrant population [3]. In Amsterdam, the respective figures for Turkish people were 5 and 15 %, respectively, and for Moroccan people 9 and 26 %, respectively [6]. Turks and Moroccans are mostly Muslim (95 %) [13].	

^a^Official statistics do not distinguish between Surinamese subgroups

^b^In the Netherlands, migrants populations are distinguished between Western (i.e., culturally proximate to the Dutch culture; migrants from, say, North-America, other European countries) and non-Western migrant populations

## Methods

### Study population

We used baseline data from the Healthy Life in an Urban Setting (HELIUS) study, a multi-ethnic cohort study in Amsterdam, the Netherlands. The full study protocol is described elsewhere [2]. Briefly, participants aged 18–70 years were randomly sampled stratified by ethnic origin through the municipality register of Amsterdam. This register includes data on the country of birth of residents and their parents, which were used to determine ethnicity (see below). We were able to contact about 65 % of those invited (Surinamese 73 %, Ghanaians 69 %, Turks 60 %, Moroccans 66 %), either by response card or after home visit by an ethnically-matched interviewer. Of those contacted, about 42 % agreed to participate (Surinamese 43 %, Ghanaians 50 %, Turks 34 %, Moroccans 32 %). After positive response, participants received a digital or paper version of the questionnaire (depending on the preference). Participants who were unable to complete the questionnaire themselves were offered assistance from a trained ethnically-matched interviewer. Data collection was still on going at the time of data analysis for this study. Written informed consent was obtained from all participants prior to the study inclusion.

Baseline data were collected from January 2011 until June 2014. From the total sample (*n* = 14,628), we excluded ethnic Dutch (*n* = 2192). From the remaining ethnic minority groups, we excluded Surinamese with Indonesian origin (*n* = 148) and with unknown origin (*n* = 151), and those with unknown ethnic background (*n* = 29), as these groups were relatively small. Subsequently, participants were excluded with missing data on PED, depression and/or education (*n* = 328). This finally resulted in 11,780 participants: 2501 South-Asian Surinamese, 2292 African Surinamese, 1877 Ghanaians, 2626 Turks, and 2484 Moroccans.

### Variables

#### Ethnicity

Participant’s ethnicity was defined according to the country of birth of the participants as well as that his parents [36]. Specifically, a participant was considered of non-Dutch ethnicity if either of the following criteria was fulfilled: (1) born outside the Netherlands and at least one parent born outside the Netherlands (i.e., first generation); or (2) born in the Netherlands, but both parents born outside the Netherlands (i.e., second generation). In addition, self-reported ethnicity was used to determine Surinamese subgroups (either African or South-Asian origin).

#### Perceived ethnic discrimination

PED was conceptualized as the day-to-day experiences of unfair treatment (both overt and subtle) because of ethnic background [9]. To measure PED we used the Everyday Discrimination Scale (EDS), a widely used scale in US studies [37, 38]. The EDS is developed based on a qualitative study among African American women, but also in African Surinamese women in the Netherlands [16], suggesting that the EDS can be used among ethnic minority groups in European settings as well. The EDS captures the frequency of experiences of discrimination in everyday life, using nine items (e.g., “being treated with less respect than others”). We adapted the EDS such that the participants were specifically asked about discriminatory experiences because of their ethnic background. The response scale for each item varied from 1 (never) to 5 (very often), consistent with the study by Forman et al. [37]. The mean discrimination score of the nine items was calculated (1 = lowest, 5 = highest) and used in the analyses. The Cronbach’s alpha was 0.91 for South-Asian Surinamese, 0.90 for African Surinamese, 0.91 for Ghanaians, 0.90 for Turks, and 0.92 for Moroccans.

#### Depressive symptoms

Depressive symptoms were assessed using the Patient Health Questionnaire-9 (PHQ-9) [39]. The PHQ-9 assesses the presence of depressive symptoms over the preceding 2 weeks. Baas et al. demonstrated the validity of PHQ-9 among Surinamese in the Netherlands [40]. The PHQ-9 consists of nine items, with a response scale varying from 0 (never) to 3 (nearly every day). Hence the total sum score for depression symptoms varied between 0 (lowest) and 27 (highest). The Cronbach’s alpha was 0.92 for South-Asian Surinamese, 0.86 for African Surinamese, 0.87 for Ghanaians, 0.90 for Turks and 0.88 for Moroccans.

#### Educational level

Educational level was defined as the highest level of education completed with a diploma or certificate of proficiency, either in the Netherlands or in the country of origin. Based on the highest level of education completed, participants were divided into four categories: no education or elementary education; lower vocational and general secondary education; intermediate vocational and higher secondary education; and higher vocational education and university.

#### Ethnic identity

Ethnic identity was conceptualized as the sense of belonging to one’s own ethnic group that shares cultural values and beliefs [41, 42]. It reflects a sense of membership and the positive feelings toward one’s ethnic heritage and/or identity [21, 42]. It was measured using the 10 items of Psychological Acculturation Scale (PAS; e.g., “I have a lot in common with Surinamese/Ghanaian/Turkish/Moroccan people”, “I feel proud to be part of Surinamese/Ghanaian/Turkish/Moroccan culture”) [43]. We did not use the single self-identification item (e.g., “I feel Surinamese”), since it may fail to fully capture the multifaceted nature of ethnic identity, as conceptualized above [42]. A multidimensional scale as the PAS might work better as a measure for ethnic identity. For example, individuals might not necessarily identify themselves as Surinamese or Ghanaian, but psychologically they could be strongly connected to and find comfort within their own group. Furthermore, ethnic minority members may provide social desirable answers to the self-identification item. We may reduce this potential bias using a multi-item scale to assess ethnic identity. The response scale of PAS ranged from 1 (totally disagree) to 5 (totally agree), leading to a sum score varying between 10 (weakest ethnic identity) and 50 (strongest ethnic identity). For assessing effect modification, we decided to dichotomize ethnic identity after considering the sum score distribution within each ethnic minority group: <40 (weak ethnic identity) and ≥40 (strong ethnic identity). The Cronbach’s alpha was 0.93 for South-Asian Surinamese, 0.92 for African Surinamese, 0.94 for Ghanaians, 0.93 for Turks, and 0.92 for Moroccans.

#### Religion

Religion was conceptualized as currently practicing religion. It was measured using a single item, “Do you practice a specific religion right now?”, with yes/no response.

#### Ethnic social network

Ethnic social network was conceptualized as the presence of same-ethnic people within one’s social network [21]. We assessed ethnic social network using two proxy items on a 5-point Likert scale: (1) “I have Surinamese/Ghanaian/Turkish/Moroccans friends” (1 = none, 5 = very many), and 2) “I spend my free time with Surinamese/Ghanaian/Turkish/Moroccan people” (1 = never, 5 = always). We initially developed a composite variable, but since the Cronbach’s alpha was low in the ethnic minority groups (varying between 0.52 and 0.71), we decided to analyse these two variables separately. One variable concerned the number of same-ethnic friends and the other was related to leisure time spent with same-ethnic people. To test effect modification, we dichotomized both variables. Based on the score distributions, both items were dichotomized at the score of 4, with score of 4 or higher indicating high number of same-ethnic friends and often (or always) spending leisure time with same-ethnic people.

### Statistical analysis

To handle missing data for PED, depression and ethnic identity, we employed the following strategy: if one of the items was missing, the mean score of the other eight items was used to replace the missing item. If more than one item was missing, the variable was considered missing. Data for ethnic identity, religion, and two ethnic social network measures were missing in less than 1 % of all participants, with little between-group variation (see also Table 1). These participants were excluded from the analyses that included these variables.

**Table 1:** Characteristics of the study population

Variable	Total sample (*n* = 11,780)	South-Asian Surinamese (*n* = 2501)	African Surinamese (*n* = 2292)	Ghanaians (*n* = 1877)	Turks (*n* = 2626)	Moroccans (*n* = 2484)
Male (%)	41.6 (4897)	45.7 (1143)	37.3 (856)	40.9 (767)	45.7 (1200)	37.5 (931)
Age in years, mean (SD)	43.01 (13.07)	45.27 (13.40)	46.94 (12.83)	44.61 (11.41)	39.80 (12.36)	39.30 (13.10)
First generation migrant, % (*n*)	77.4 (9116)	77.3 (1933)	83.0 (1903)	94.2 (1768)	70.3 (1846)	67.1 (1666)
Education, % (*n*)^a^						
1 (lowest)	23.5 (2773)	15.4 (384)	6.8 (156)	29.5 (554)	33.5 (880)	32.2 (799)
2	29.5 (3471)	33.8 (846)	34.4 (789)	38.7 (726)	24.9 (655)	18.3 (455)
3	30.5 (3590)	29.2 (731)	35.9 (822)	25.4 (476)	28.0 (735)	33.3 (826)
4 (highest)	16.5 (1946)	21.6 (540)	22.9 (525)	6.4 (121)	13.6 (356)	16.3 (404)
Perceived ethnic discrimination score, mean (SD), range 1 (lowest)–5 (highest)	1.92 (0.76)	1.97 (0.75)	2.01 (0.76)	1.87 (0.79)	1.82 (0.73)	1.96 (0.79)
Depressive symptoms score, mean (SD), range 0 (lowest)–27 (highest)	5.03 (5.50)	5.36 (5.79)	3.90 (4.52)	3.33 (4.33)	6.24 (6.01)	5.75 (5.75)
Ethnic identity^b^, % (*n*)						
Strong ethnic identity (score ≥ 40)	65.9 (7716)	51.8 (1290)	63.0 (1435)	76.8 (1432)	74.7 (1944)	65.5 (1615)
Missing	0.7 (78)	0.4 (10)	0.6 (13)	0.7 (13)	0.8 (22)	0.8 (20)
Religion, % (*n*)						
Currently practicing	88.2 (10,302)	81.1 (2019)	78.1 (1785)	88.2 (1608)	94.0 (2448)	98.6 (2442)
Missing	0.8 (97)	0.4 (10)	0.3 (6)	2.8 (53)	0.8 (21)	0.3 (7)
Number of same-ethnic friends^c^, % (*n*)						
Many (score ≥ 4)	47.3 (5527)	29.3 (728)	44.0 (1000)	64.2 (1196)	62.7 (1623)	39.7 (980)
Missing	0.9 (107)	0.7 (18)	0.8 (18)	0.8 (15)	1.4 (38)	0.7 (18)
Leisure time with same-ethnic people^d^, % (*n*)						
Often/always (score ≥ 4)	57.8 (6756)	51.0 (1268)	63.0 (1435)	59.9 (1113)	61.2 (1582)	54.9 (1358)
Missing	0.8 (97)	0.5 (13)	0.6 (14)	1.0 (19)	1.5 (40)	0.4 (11)

*SD* standard deviation

^a^1 = no education or elementary education; 2 = lower vocational and general secondary education; 3 = intermediate vocational and higher secondary education; 4 = higher vocational education or university

^b^Ethnic identity: sum score ranges from 10 to 50, with a cut-off at 40 (<40 weak ethnic identity, ≥40 strong ethnic identity)

^c^Same-ethnic friends: score range 1–5, with a cut-off at 4 (<4 low number, ≥4 high number)

^d^Leisure time spent score range 1–5, with a cut-off at 4 (<4 sometimes, ≥4 often/always)

Linear regressions were used to examine the association between PED and depressive symptoms. The normal probability plot (P–P plot) of residuals of depressive symptoms showed that this variable was about normally distributed. The regressions were estimated for the total sample and for each ethnic minority group separately. We performed the analyses with the total sample to increase the statistical power and to possibly identify any interaction effects which may be too small to be demonstrated in the group-specific analysis. The models were adjusted for ethnicity (only in the total sample), sex, age, migration generation, and education. In a previous study using similar data we found that these potential confounders attenuated the association between PED and depressive symptoms [44]. To assess whether this association differed by ethnicity, we used the interaction term (PED*Ethnicity). To assess whether ethnic identity, religion, and ethnic social network measures modified the association, we created interaction terms of the psychosocial factor and PED (e.g., religion*PED). SPSS version 21.0 was used for analysis.

## Results

Characteristics of the study population are presented in Table 1. The majority of the ethnic minority groups was first-generation. Average age for both Surinamese subgroups was 45 years, and for Turks and Moroccans around 40 years. Ghanaians and Turks more often had a lower education while the Surinamese subgroups had medium education. Mean PED scores were largely similar across the ethnic minority groups, with a mean score around 2. Depressive symptoms were more common in South-Asian Surinamese, Turks, and Moroccans and less so in African Surinamese and Ghanaians.

Most Turks and Ghanaians had a strong ethnic identity (around 75 %), and for South-Asian Surinamese this was around 50 %. The majority of the participants practiced religion, particularly Turks and Moroccans (around 95 %). Above 60 % of Turks and Ghanaians had many same-ethnic friends and often spent leisure time with same-ethnic people.

Table 2 shows the association between PED and depressive symptoms and the buffering effects of the psychosocial factors. In all ethnic minority groups, PED was positively associated with depressive symptoms, after adjusting for sex, age, migration generation, and education. The association differed by ethnicity (*p* value for interaction 0.001). For example, the association was stronger in South-Asian Surinamese [regression coefficient 1.88; 95 % confidence interval (CI) 1.59–2.17] than in African Surinamese (1.21; 0.98–1.45).

**Table 2:** The association between perceived ethnic discrimination (PED) and depressive symptoms according to potential effect modifiers

Stratified by	Adjusted regression coefficient (95 % CI) for the association PED-depressive symptoms					
	Total sample	South-Asian Surinamese	African Surinamese	Ghanaians	Turks	Moroccans
Association^a^ without interaction	1.68 (1.55, 1.80)	1.88 (1.59, 2.17)	1.21 (0.98, 1.45)	1.63 (1.40, 1.87)	2.11 (1.80, 2.41)	1.51 (1.23, 1.80)
Ethnic identity						
Weak	1.86 (1.65, 2.07)	2.00 (1.58, 2.43)	1.64 (1.26, 2.02)	2.00 (1.51, 2.48)	2.16 (1.60, 2.71)	1.53 (1.05, 2.00)
Strong	1.51 (1.36, 1.66)	1.76 (1.36, 2.16)	0.90 (0.61, 1.19)	1.48 (1.21, 1.75)	2.00 (1.63, 2.36)	1.37 (1.02, 1.72)
*p* value for interaction	0.008*	0.414	0.002*	0.070	0.638	0.599
Religion						
No	1.63 (1.26, 2.00)	1.85 (1.18, 2.52)	1.61 (1.10, 2.12)	1.07 (0.34, 1.79)	1.36 (0.10, 2.63)	4.43 (1.92, 6.95)
Yes	1.68 (1.55, 1.81)	1.89 (1.57, 2.21)	1.11 (0.84, 1.37)	1.69 (1.44, 1.95)	2.14 (1.83, 2.46)	1.47 (1.18, 1.76)
*p* value for interaction	0.808	0.926	0.085	0.112	0.241	0.022*
Number of same-ethnic friends						
Low	1.84 (1.68, 2.01)	2.15 (1.80, 2.50)	1.20 (0.88, 1.51)	1.98 (1.59, 2.37)	2.50 (2.01, 2.99)	1.57 (1.20, 1.94)
High	1.47 (1.29, 1.65)	1.11 (0.57, 1.65)	1.21 (0.86, 1.56)	1.38 (1.08, 1.67)	1.87 (1.48, 2.26)	1.44 (1.00, 1.88)
*p* value for interaction	0.004*	0.001*	0.958	0.016*	0.046*	0.653
Leisure time with same-ethnic people						
Sometimes	1.91 (1.72, 2.09)	1.96 (1.56, 2.37)	1.76 (1.38, 2.14)	1.56 (1.19, 1.92)	2.46 (1.98, 2.93)	1.75 (1.33, 2.16)
Often/always	1.49 (1.33, 1.66)	1.78 (1.36, 2.20)	0.90 (0.61, 1.19)	1.66 (1.35, 1.98)	1.92 (1.52, 2.32)	1.31 (0.92, 1.69)
*p* value for interaction	0.001*	0.535	<0.001*	0.669	0.087	0.128

Analyses in the total sample and per ethnic group

*CI* confidence interval

* Denotes statistical significant interaction term at the *p* < 0.05 level with two-tailed test

^a^This association was adjusted for ethnicity (in the total sample only), sex, age, migration generation, and education. All associations in the interaction analyses were adjusted for these variables as well

### Ethnic identity

Ethnic identity buffered the association between PED and depressive symptoms in the total sample (Table 2). Those with a strong identity had a regression coefficient of 1.51 (95 % CI 1.35–1.66) versus 1.86 (1.65–2.07) with weak ethnic identity (*p* value for interaction 0.008). This buffering effect was particularly observed in African Surinamese (strong ethnic identity 0.90; 0.61–1.19 versus weak ethnic identity 1.64; 1.26–2.02), and to lesser extent in Ghanaians (1.48; 1.21–1.75 versus 2.00; 1.51–2.48). No buffering effects were observed in the other ethnic minority groups.

### Religion

Religion did not weaken the association between PED and depressive symptoms in the total sample, but the pattern differed by ethnic minority group. The association was weaker among those who practice religion in Moroccans (regression coefficient 1.47; 95 % CI 1.18*–*1.76 versus not being religious 4.43; 1.91*–*6.95) and to lesser extent in African Surinamese (1.11; 0.84*–*1.37, compared to not being religious 1.61; 1.10*–*2.12).

### Same-ethnic friends

Having many same-ethnic friends weakened the association between PED and depressive symptoms in the total sample (regression coefficient 1.47; 95 % CI 1.29–1.65 versus low number of same-ethnic friends 1.84; 1.68–2.01). This effect was most pronounced in South-Asian Surinamese (1.11; 0.57–1.65 versus 2.15; 1.80–2.50). A similar pattern was also observed among Turks and Ghanaians.

### Leisure time with same-ethnic people

In the total sample, the association between PED and depressive symptoms was weaker among those who often spend leisure time with same-ethnic people (1.49; 1.33–1.66 versus spend sometimes 1.91; 1.72–2.09). The effect was found particularly among African Surinamese (0.90; 0.61–1.19 versus 1.76; 1.38–2.14) and less so in Turks (1.92; 1.52–2.32 versus 2.46; 1.98–2.93). No buffering effects were observed in the other ethnic minority groups.

## Discussion

This study found that perceived ethnic discrimination (PED) was positively associated with depressive symptoms in ethnic minority groups. Ethnic identity, religion, and ethnic social network weakened this association, although the buffering effects differed by ethnic minority group. We observed that the association between PED and depressive symptoms was weaker among (a) those with a strong ethnic identity in African Surinamese and Ghanaians, (b) those who practice religion among African Surinamese and Moroccans, and (c) those with a large ethnic social network in all ethnic minority groups (except Moroccans).

This study had some potential limitations. First, the design of this study was cross-sectional, thus limiting the possibilities for causal inferences. However, we were mainly interested in exploring possible effect modification by the psychosocial factors, and not necessarily in the association between PED and depressive symptoms as such. Second, the sample consisted of populations living in the one European city, therefore the findings may not be generalised to other European cities. Third, the response rates were quite low, so non-response bias might have occurred. Since our study mainly focused on the interaction analyses and not prevalence estimates, the selective response may not necessarily be an important limitation. The selective response might have biased the prevalence estimates, but is seems unlikely that it may have biased the strength and direction of the interaction effects. Finally, the measurement of the psychosocial factors might not be adequate enough to fully capture the buffering effects in relation to PED. For example, for ethnic social network we only assessed the quantitative aspect, but not the qualitative. Religion was measured with a single question, but could have been supplemented with, say, religious social support and type of religion. A study among Arab Americans showed that the association between ethnic discrimination and psychological distress tended to be stronger among Christians than Muslims [45].

We found evidence suggesting that the buffering effect of ethnic identity was strongest in African Surinamese and to lesser extent Ghanaians. Maybe for these two African-origin groups, particularly for African Surinamese, the health-buffering effect of ethnic identity operates through sense of belonging, which might be related to their socio-cultural history of slavery and racism [46]. And because of this historical awareness, ethnic identity could be an important source of resilience for these particular groups to overcome the effects of PED. For the other ethnic minority groups, different dimensions of ethnic identity may act as buffers in relation to PED (e.g., family history, belonging to the ethnic community).

We further found that religion weakened the association between PED and depressive symptoms in African Surinamese and particularly in Moroccans, but not in other groups. This is only in part consistent with previous studies from the US, which showed that religion had protective effect in African Americans [31], but no effect in Arab Americans [45]. Our divergent pattern across ethnic minority groups could possibly be explained by how religion is experienced, and how it relates to discrimination. Maybe among African Surinamese and Moroccans religion is a positive phenomenon (e.g., source of strength or inspiration, social support), making them resilient in the face of daily stressors [29]. A Dutch report indicated that Moroccans are more actively engaged in religious activities, partly as a response to the currently hostile climate toward their Muslim-Moroccan background [13]. Interestingly, only 2 % of Moroccans did not practice religion and they could differ from those who are religious in different ways. For example, they could experience more overall discrimination, both by other-group members (because of their ethnic background) and by same-group members (social exclusion due to religious abandonment).

The measures of ethnic social network tend to have buffering effects in most ethnic minority groups. Among most ethnic minority groups, same-ethnic friends had protective effect, suggesting that same-ethnic friends might serve for them as an outlet to share their discriminatory experiences to reduce the psychological burden. In African Surinamese, however, spending leisure time with same-ethnic people was protective. It could be that for African Surinamese experiencing discrimination, given its historical connotation, is being seen as personal failure or shameful [16], so people might be cautious in discussing such experiences with their friends, but rather engage in ethnic social activities (including those organized by the church).

It could be argued that the buffering effects of ethnic social network, as found in this study, owes to the social network in general, regardless of the ethnic nature. However, in additional analyses (data not shown), we did not find any evidence that having ethnic Dutch friends and spending leisure time with ethnic Dutch people weakened the association between PED and depressive symptoms across the ethnic minority groups.

In conclusion, ethnic identity, religion, and ethnic social network weakened the association between PED and depressive symptoms, but the buffering effects differed by ethnic minority group. The particular psychological and sociological meanings different ethnic minority groups attach to these certain psychosocial factors might help understand these disparate buffering effects. Further research should investigate the effects of ethnic identity, religion, and ethnic social network in relation to PED in more depth, both quantitatively and qualitatively. Quantitative studies could explore the different dimensions of, say, religion such as the frequency of attending religious services, social support from religious institutions. Qualitative research could unravel how ethnic minority groups use and which meaning they attach to various psychosocial factors in relation to PED. This may help to grasp the underlying sources of resilience that are employed by ethnic minority groups to overcome experiences of ethnic discrimination.
